# Dosimetric impact of rotational errors on the quality of VMAT‐SRS for multiple brain metastases: Comparison between single‐ and two‐isocenter treatment planning techniques

**DOI:** 10.1002/acm2.12815

**Published:** 2020-02-05

**Authors:** Georgia Prentou, Eleftherios P Pappas, Andreas Logothetis, Efi Koutsouveli, Evaggelos Pantelis, Panagiotis Papagiannis, Pantelis Karaiskos

**Affiliations:** ^1^ Medical Physics Laboratory Medical School National and Kapodistrian University of Athens Athens Greece; ^2^ Medical Physics Department Hygeia Hospital Athens Greece

**Keywords:** brain metastases, rotational error, single isocenter, spatial uncertainty, stereotactic radiosurgery, VMAT

## Abstract

**Purpose:**

In the absence of a 6D couch and/or assuming considerable intrafractional patient motion, rotational errors could affect target coverage and OAR‐sparing especially in multiple metastases VMAT‐SRS cranial cases, which often involve the concurrent irradiation of off‐axis targets. This work aims to study the dosimetric impact of rotational errors in such applications, under a comparative perspective between the single‐ and two‐isocenter treatment techniques.

**Methods:**

Ten patients (36 metastases) were included in this study. Challenging cases were only considered, with several targets lying in close proximity to OARs. Two multiarc VMAT plans per patient were prepared, involving one and two isocenters, serving as the reference plans. Different degrees of angular offsets at various orientations were introduced, simulating rotational errors. Resulting dose distributions were evaluated and compared using commonly employed dose‐volume and plan quality indices.

**Results:**

For single‐isocenter plans and 1^⁰^ rotations, plan quality indices, such as coverage, conformity index and D_95%_, deteriorated significantly (>5%) for distant targets from the isocenter (at> 4–6 cm). Contrarily, for two‐isocenter plans, target distances to nearest isocenter were always shorter (≤4 cm), and, consequently, 1^⁰^ errors were well‐tolerated. In the most extreme case considered (2^⁰^ around all axes) conformity index deteriorated by on‐average 7.2%/cm of distance to isocenter, if one isocenter is used, and 2.6%/cm, for plans involving two isocenters. The effect is, however, strongly associated with target volume. Regarding OARs, for single‐isocenter plans, significant increase (up to 63%) in D_max_ and D_0.02cc_ values was observed for any angle of rotation. Plans that could be considered clinically unacceptable were obtained even for the smallest angle considered, although rarer for the two‐isocenter planning approach.

**Conclusion:**

Limiting the lesion‐to‐isocenter distance to ≤4 cm by introducing additional isocenter(s) appears to partly mitigate severe target underdosage, especially for smaller target sizes. If OAR‐sparing is also a concern, more stringent rotational error tolerances apply.

## 1 INTRODUCTION

Stereotactic radiosurgery (SRS) is a well‐established radiotherapy technique for the treatment of a variety of lesions, mainly in the brain.^1^, ^2^, ^3^ Regarding the management of multiple brain metastases, SRS is being increasingly employed even in cases with more than 10 lesions.^4^, ^5^ However, increased conformity and presence of steep dose gradients in SRS treatment plans demand increased spatial accuracy in order to ensure effective treatment delivery, as spatial errors of just a few millimeters can induce considerable target underdosage, especially in tiny brain lesions.^6^, ^7^, ^8^

Volumetric modulated arc therapy (VMAT) is commonly employed for SRS treatment delivery. Several studies have demonstrated that multiarc noncoplanar VMAT can deliver highly conformal plans to the target(s) and spare adjacent critical structures.^9^, ^10^, ^11^, ^12^, ^13^, ^14^, ^15^, ^16^ More recently, single‐isocenter VMAT‐SRS treatment techniques were introduced for dose delivery to multiple intracranial targets/lesions concurrently, with the latter being an attractive approach since treatment duration can be further reduced without necessarily compromising plan quality.^17^, ^18^, ^19^ A single isocenter has been found sufficient for VMAT‐SRS of multiple intracranial metastases, whereas minor improvements in plan quality can be achieved when additional isocenter(s) are used.^20^

The main drawback of a single‐isocenter VMAT‐SRS technique is that it exhibits increased sensitivity to geometric uncertainties (compared to other approaches) and, therefore, its efficacy partly relies on the overall spatial accuracy.^9^, ^12^, ^17^, ^18^, ^21^, ^22^, ^23^, ^24^ Patient positioning and immobilization is a typical source of translational and rotational uncertainties.^25^ Thermoplastic masks are commonly used in intracranial frameless VMAT‐SRS applications, and residual patient setup errors can be detected using appropriate image‐guided techniques.^25^ Translational setup errors are easily corrected for by adapting the treatment couch position. However, initial rotational errors can be accounted for only if a 6 degree‐of‐freedom (DOF) robotic couch is available, which is not always the case.^26^, ^27^, ^28^ Nevertheless, regardless of pretreatment imaging and initial setup correction methods, significant intrafractional patient motion (including rotations) has been repeatedly reported for intracranial VMAT‐SRS cases.^26^, ^28^, ^29^, ^30^, ^31^

In addition to patient positioning, other potential sources of rotational errors cannot be ruled out. For instance, the magnetic resonance imaging (MRI)‐computed tomography (CT) spatial coregistration procedure could contribute to the overall spatial uncertainty budget. For a cranial case, the MR/CT registration uncertainty was estimated at 1.8 mm in a multi‐institutional study.^32^ Although rotational uncertainties were not separately reported, it can be expected that they may considerably contribute to the overall spatial uncertainty, especially for targets lying away from the MR isocenter where MR images inherently exhibit increased geometric warping.^33^, ^34^, ^35^ Furthermore, geometric uncertainties stemming from the linac rotating parts (i.e., gantry, collimator or couch) or related to the angular alignment accuracy between the (i) on‐couch imaging system (kV or MV CT), (ii) mechanical, and (iii) radiation delivery isocenters should also be taken into consideration.^36^, ^37^ Rotational errors are more important in single‐isocenter multitarget cases, as lesions may lie several centimeters away from the isocenter and, therefore, induce considerable translations. As an instance, by performing off‐axis Winston‐Lutz tests, it was recently shown that radiation and on‐couch imaging isocenters mis‐alignment can induce offsets up to 1 mm at a distance of 60 mm from the isocenter.^36^

Acknowledging the importance of spatial accuracy, several studies have investigated the dosimetric effect of rotational errors on linac‐based SRS for brain metastases cases, mostly focusing on target/lesion underdosage and the potentially induced loss of coverage.^29^, ^30^, ^31^, ^38^ However, in all of the above studies the corresponding dosimetric impact on organs at risk (OARs) was not examined. In a recent study, Sagawa et al.^39^ studied the dose‐increase to the normal brain parenchyma but disregarded the effect on other critical structures such as the brainstem and the optic pathway. To our knowledge, the only work reporting on OAR‐sparing focused on single‐target cranial SRS, where a small rotational error was found to have a significant dosimetric impact in cases with OARs in close proximity to the target volume.^24^ In multitarget single‐isocenter VMAT‐SRS, due to the off‐axis locations of targets and the steep dose gradients employed for sparing an adjacent OAR (e.g., the optic pathway), even a small geometric tilt could also result in significant over‐dosage to the OAR, especially when the adjacent off‐axis target is also located away from the isocenter, which is not uncommon in multitarget single‐isocenter SRS. Although not evaluated in their study, Roper et al. commented that the potential of rotational errors to overdose normal tissues is an important clinical concern and for lesions in close proximity to critical structures (e.g., optic nerves, chiasm, or brainstem), setup errors that result in collateral damage to these adjacent structures may be as critical as setup errors that underdose a target and, thus, require further investigation.^17^

The scope of the present work was to study the dosimetric impact of rotational errors on target coverage and OAR‐sparing in multitarget VMAT‐SRS brain metastases cases, focusing on cases with OARs lying in close proximity to targets, located at various distances from the isocenter(s). Tolerance to rotational errors is studied and quantified. Single‐ and two‐isocenter treatment plans are both considered and compared in order to investigate the potential benefit of reduced risk if two isocenters are used, in contrast to the shorter treatment time and minimum setup effort associated with the single‐isocenter technique. Toward that end, using both techniques, reference plans are created for 10 patients referred to for 3 or 4 brain metastases (a total of 36 lesions). Although an in‐depth comparison between reference plans (i.e., planning techniques) is beyond the scope of this work, single‐ and two‐isocenter plans are both presented and evaluated using common dose‐volume and plan quality metrics employed in SRS clinical practice. Furthermore, rotational errors are simulated by rotating the calculated reference dose distributions up to 2°, with the isocenter(s) serving as the origin(s). Induced geometric shifts are calculated. Dosimetric impact on both targets and OARs is quantified and associated with distance to the nearest isocenter.

## 2 METHODS

### 2.1 Targets and OARs delineation

Ten challenging cases were retrospectively included in this study. In particular, effort was made to involve cases of up to four small metastases each (approximately 1–2 cm in diameter) and critical structures lying in close proximity to targets. Moreover, increased intralesion distances were also a suitable characteristic for the purposes of this study. The corresponding treatment planning cranial CT datasets and contours were anonymized and imported in Monaco version 5.10, (ELEKTA, Crawley, UK) treatment planning system (TPS) for treatment planning and dose calculations.

**Figure 1:**
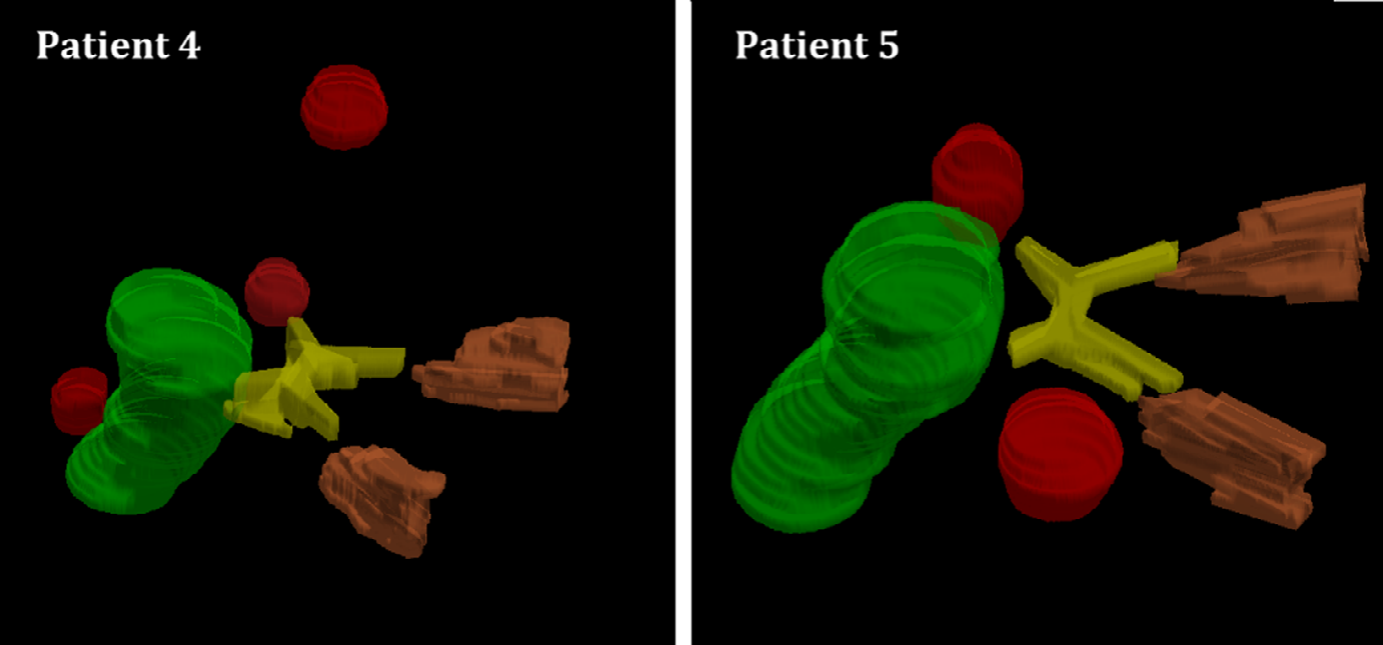
A 3D illustration of selected targets lying in close proximity to OARs (brainstem, optic chiasm, optic nerves) for two indicative cases. Contours legend: targets: red, brainstem: green, optic chiasm: yellow, optic nerves: brown.

The selected cases involved either three or four metastases (related to four and six patients, respectively), located in the brain parenchyma with at least one target lying in close proximity to OAR(s) (as indicatively shown in Fig. 1) (minimum target‐to‐OAR distance of approximately 0.5 cm). Other targets (not shown in Fig. 1) were more distant from critical structures or other targets, that is, resulted to increased interlesion distances. Details of the contoured targets are given in Table 1. In all selected cases, patients had been positioned in a Head‐First Supine (HFS) position.

**Table 1:** Summary of physical characteristics related to all 36 targets, including distances to the nearest isocenter if the (a) single‐ and (b) two‐isocenter planning technique is employed.

Physical characteristic	Min	Max	Median
Diameter (cm)	0.96	2.11	1.50
Volume (cc)	0.46	4.42	1.99
(a) Distance to isocenter (cm)	1.95	6.55	4.47
(b) Distance to nearest isocenter (cm)	0.00	4.04	2.97

In order to better serve the scope of this study, effort was made to involve a wide range of lesion‐to‐isocenter distances, whether a single‐ or a two‐isocenter plan (see section 2.B) is created. The resulting lesion‐to‐isocenter distances are also given in Table 1. Since the simulated rotational errors occurred with respect to the plan’s isocenter, it is geometrically expected that the induced spatial offset will be more enhanced at target locations distant from the isocenter.^17^, ^30^

### 2.2 Reference treatment plans

**Figure 2:**
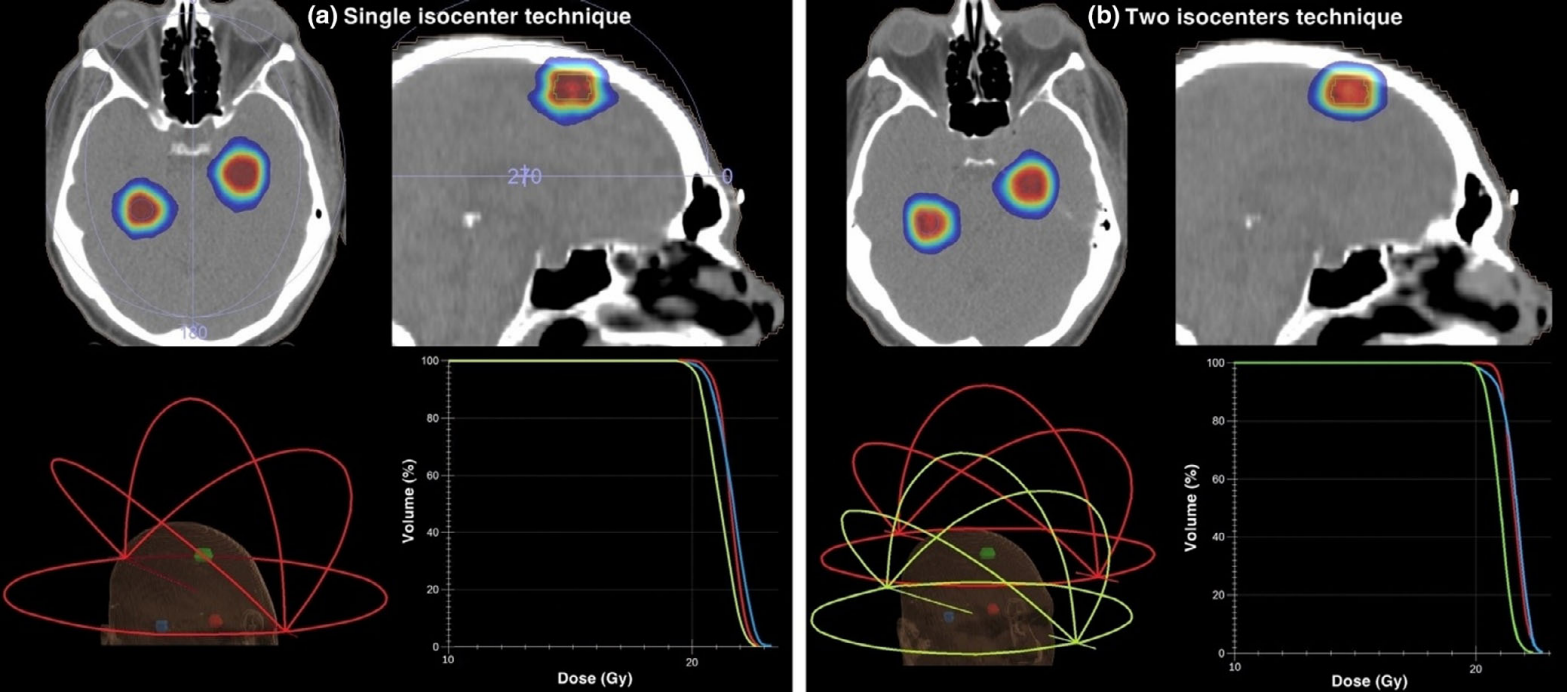
Indicative (patient #4) reference treatment plans prepared using both the (a) single‐ and (b) two‐isocenter techniques. Isodose lines, corresponding to reference dose distributions, are superimposed on axial and sagittal slices (top) of the planning CT scan. Arcs configuration and related DVHs are also presented (bottom).

Treatment planning was performed using the Monaco TPS. Noncoplanar VMAT‐SRS plans were prepared for all ten cases with the following arc arrangement: a 360° arc (couch angle: 0º) and three half arcs (couch angles: 45º, 90º, 315º). Arcs configuration is graphically illustrated in Fig. 2. An Agility linear accelerator (ELEKTA, Crawley, UK) with a 5‐mm MLC, and 6MV flattening‐filter‐free (FFF) beams was used. For all cases and targets, a dose of 20 Gy was prescribed in a single dose fraction. All dose calculations were performed using the X‐Ray Voxel Monte Carlo (XVMC) dose calculation algorithm with a uniform dose calculation grid resolution of 1 mm.^40^

For each patient, two plans were prepared. The first approach involved a single isocenter with its location defined by the geometric center of all targets considered. In order to prioritize high target coverage, planning goals assured V_20Gy_ ≥ 98%, that is, the prescription isodose covers 98% of each target volume. During plan optimization, the clinical procedure was followed in order to achieve the intended planning goals such as high‐dose conformity and steep dose gradients. Regarding OARs, the dose criteria given in Table 2 were considered and strictly met in all cases. The aforementioned planning method has been repeatedly implemented in other independent studies.^11^, ^13^, ^14^

**Table 2:** Dose constraints strictly applied to OARs during reference treatment planning, for all cases considered.

Structure	Metric	Constraint
Brainstem	D_max_	≤15 Gy
Optic nerve	D_max_	≤8 Gy
Optic chiasm	D_max_	≤8 Gy
Lens	D_max_	≤1 Gy
Brain	V_7Gy_	≤6%
	V_12Gy_	≤30 cc

Abbreviations: D_max_, maximum dose; OARs, organs at risk; V_7Gy_, percentage volume of structure receiving at least 7 Gy; V_12Gy_, absolute volume of structure receiving at least 12 Gy.

For the comparison purposes of this study, two‐isocenter plans were also prepared with all other planning and calculation parameters kept constant. However, isocenter positioning followed a different approach in order to involve two isocenters. Regarding cases of four brain metastases, the first  isocenter was placed at the geometric center of the two targets lying closest to each other, whereas the second isocenter was positioned at the geometric center of the remaining two targets. In a similar way, for cases with three brain metastases (indicatively shown in Fig. 2), the first isocenter was placed at the geometric center of the two closest targets and the second one at the center of the remaining target. In this way the maximum lesion‐to‐isocenter distance was limited to 4 cm, for the patients included in this study, in contrast to corresponding distances of up to 6.55 cm occurring for plans of one isocenter (Table 1). Each isocenter was associated with the same four noncoplanar arcs as the ones considered for the single‐isocenter planning approach, with MLCs and jaws collimated to include only the respective target(s).^20^ In all cases, the two isocenters were optimized simultaneously using the same optimization criteria as with the single‐isocenter plans.

### 2.3 Rotational errors simulation

In order to simulate and estimate the dosimetric effect of rotational errors, the reference dose distributions (corresponding to the reference plans, section 2.B) were rotated around the plan isocenter(s). To accomplish that, planning data were exported from the TPS in DICOM RT format and imported to MATLAB^®^ (The MathWorks, Inc, Natick, MA). An in‐house routine was developed for rotating and resampling the dose calculation grid. More specifically, rigid transformations were defined by a 3 × 3 rotation matrix and a unity translation vector. The rotated dose distributions were calculated in a new grid of the same spatial resolution and size as the reference ones. Dose values were computed into the inversely rotated new grid positions by linear interpolation. The transformed dose distributions were written in DICOM RT format for further analysis.

For the single‐isocenter treatment technique, the reference dose distribution data were rotated around the isocenter at different introduced degrees of rotation: ±0.5°, ±1°, ±2°. Angular offsets of>±2° are not often encountered in a clinical setting^31^, ^39^ and, therefore, excluded from this study. Rotations occurred around the x‐axis, y‐axis, and z‐axis independently, as well as around all three axes (DICOM coordinate system adopted throughout this study). Since all patients had been positioned in an HFS position, rotations around x, y, or z axis always correspond to pitch, yaw and roll directions, respectively. Both negative and positive rotations were applied in order to include the case where higher isodoses are shifted closer to OARs lying in close proximity to targets.

For the two‐isocenter planning technique, simulated rotations occurred around each isocenter, in order to simulate systematic rotational uncertainties. For this purpose, the reference two‐isocenter plan was divided in two sub‐plans. The first sub‐plan included the first isocenter keeping the corresponding planning parameters (beam shapes, monitor units, etc.) the same with the reference plan, whereas the other sub‐plan included the second isocenter. The dose was re‐calculated for each sub‐plan and obtained dose distributions were summed and verified that the result was identical with the corresponding reference dose distribution. Involvement of sub‐plans was essential in order to create and export two separate DICOM RT dose files, each corresponding to a different isocenter and, subsequently, simulate rotational errors similar to the procedure followed for the single‐isocenter plans. In this way, different degrees of rotation were applied using the MATLAB routine described earlier and resulting dose distributions corresponding to sub‐plans were summed in order to obtain the total dose distribution. Using this methodology, systematic rotational errors are simulated for each patient setup (i.e., isocenter) independently.

Furthermore, the spatial shifts of metastases related to rotational errors occurring around the isocenter(s) were also investigated for both treatment planning techniques. Therefore, the targets position vectors within the DICOM coordinate system were rotated using the methodologies described earlier. Induced target displacement was then calculated as the 3D Euclidian distance between the original and rotated centers of each target.

### 2.4 Plan evaluation and comparison

Reference and rotated dose distributions were analyzed and compared in MATLAB using in‐house routines or using BrachyGuide (version 2.1.0), a MATLAB‐based DICOM RT viewer, employed and validated in several previous studies.^41^, ^42^

Clinically used dose‐volume metrics for targets and OARs were calculated for both reference and rotated dose distributions, such as D_max_ (the maximum dose delivered to a structure) and V_xGy_ (the volume of a structure receiving at least x Gy). DVH analysis was performed for all structures involved. Plan conformity indices, such as Paddick’s conformity index (PCI) and gradient index (GI) were considered in this study for all dose distributions and both planning techniques.^45^, ^46^

In order to evaluate the reference plans with respect to achieved conformity, dose‐volume indices and planning goals, (serving as the reference values for the simulation study results (section 3.B)), the single‐isocenter reference plans were compared against the two‐isocenter reference dose distributions (i.e., a comparison between “zero‐rotation” plans) using the above dose‐volume and plan quality indices. Furthermore, geometric shifts induced by the simulated rotational offsets were calculated for all targets for both single‐ and two‐isocenter planning approaches. In order to investigate the dosimetric impact of rotational errors on targets and OARs, rotated dose distributions were evaluated against the corresponding reference plan for each patient.

## 3 RESULTS

### 3.1 Reference plans

The reference plans for both planning techniques (corresponding to zero rotational errors) were found clinically acceptable and typical to the ones delivered in VMAT‐SRS clinical practice. Target coverage was adequately high (V_20Gy_ > 95%) for all reference plans and cases, in‐line with the planning priorities and strategy followed. However, for the majority of targets, V_20Gy_ was higher for the two‐isocenter technique. Accordingly, PCI values (GI values, respectively) of targets were in the range of 0.61 ‐ 0.86 (4.14 ‐ 6.53), for all single‐isocenter reference plans, with corresponding values for the two‐isocenter plans slightly improved for most targets and lied in the range of 0.63–0.89 (3.65–5.78). The most distant targets from the isocenter are related to the lowest PCI values.

Dose‐volume metrics related to OARs were in agreement with plan quality criteria considered during treatment planning, although all cases were rather challenging with one or more OARs located very close to metastases (indicatively shown in Fig. 1). Consequently, D_max_ values were just below the allowed dose limits (see Table 2) for the brainstem, optic pathway and lenses. Regarding brain parenchyma, V_7Gy_ was in the range of 3.5% ‐ 6.2% for the single‐isocenter technique, whereas the corresponding range using two isocenters was reduced to 3.3% ‐ 4.9%. Accordingly, V_12Gy_ and V_13Gy_ slightly improved for the two‐isocenter reference plans. An indicative example (patient #5) of plan conformity and dose‐volume metrics for the two planning techniques is presented in Table 3.

**Table 3:** An indicative example (patient #5) of DVH metrics calculated for targets and OARs, related to the (a) single‐ and (b) two‐isocenter reference plans, for comparison purposes.

Targets	V (cc)	V_20Gy_ (%)		PCI		GI	
		(a)	(b)	(a)	(b)	(a)	(b)
meta1	2.538	96.63	98.91	0.62	0.67	5.40	4.64
meta2	2.213	99.35	99.70	0.62	0.64	5.74	4.91
meta3	2.497	98.38	99.96	0.77	0.79	4.97	4.18
meta4	3.668	98.75	99.11	0.77	0.86	4.76	3.95

OARs	V (cc)	V_7Gy_ (%)		V_12Gy_ (Gy)		V_13Gy_ (Gy)	
		(a)	(b)	(a)	(b)	(a)	(b)
Brain	1510.6	5.96	4.86	27.58	18.29	20.13	14.31

	V (cc)	D_max_ (Gy)		D_0.02cc_ (Gy)			
		(a)	(b)	(a)	(b)		
Optic Chiasm	1.732	8.00	6.80	6.16	6.00		
R optic nerve	2.179	7.60	7.20	6.10	6.54		
L optic nerve	2.204	6.00	5.00	5.32	4.78		
Brainstem	27.913	14.80	14.20	12.80	11.63		
R lens	0.443	1.00	0.85	0.93	0.77		
L lens	0.437	0.92	0.75	0.82	0.68		

Abbreviations: GI, gradient index; PCI, Paddick’s conformity index.

However, the treatment planning techniques resulted to substantially different beam‐on times, as expected. If two isocenters are used, monitor units increase by nearly 1.5‐fold which is expected to increase overall treatment duration by a factor of up to 2.

### 3.2 Simulated rotational errors

#### 3.2.1 Induced geometric offset on targets

Following simulation of angular offsets, the induced target displacements were calculated for all 36 targets involved in this study. To assist comparison between planning techniques, all results are presented in Table 4. Using two isocenters average target‐to‐isocenter distance is greatly reduced. Consequently, targets spatial offsets, induced by rotational errors, are minimized (Table 4). Median spatial offset is reduced by at least 35% compared to one isocenter plans. However, geometric displacements of > 1 mm are still noticed for both techniques, when rotational errors ≥ 1° affected targets located at distances of about 4 cm. Therefore, the geometric effect cannot be considered negligible, for large lesion‐to‐isocenter separations, irrespective of the planning technique employed.

**Table 4:** Range and median of target displacements induced by simulated rotational errors for the (a) single‐ and (b) two‐isocenter techniques.

	Range (mm)		Median (mm)	
	(a)	(b)	(a)	(b)
single axis rotations				
±0.5°	0.05–0.57	0.00–0.39	0.36	0.21
±1°	0.08–1.12	0.00–0.70	0.68	0.43
±2°	0.16–2.29	0.00–1.47	1.30	0.85
three axes rotations				
± 0.5°	0.21–0.98	0.00–0.63	0.69	0.38
± 1°	0.39–1.93	0.00–1.23	1.31	0.74
± 2°	0.84–3.89	0.00–2.58	2.58	1.46

#### 3.2.2 Dosimetric effect on targets

**Figure 3:**
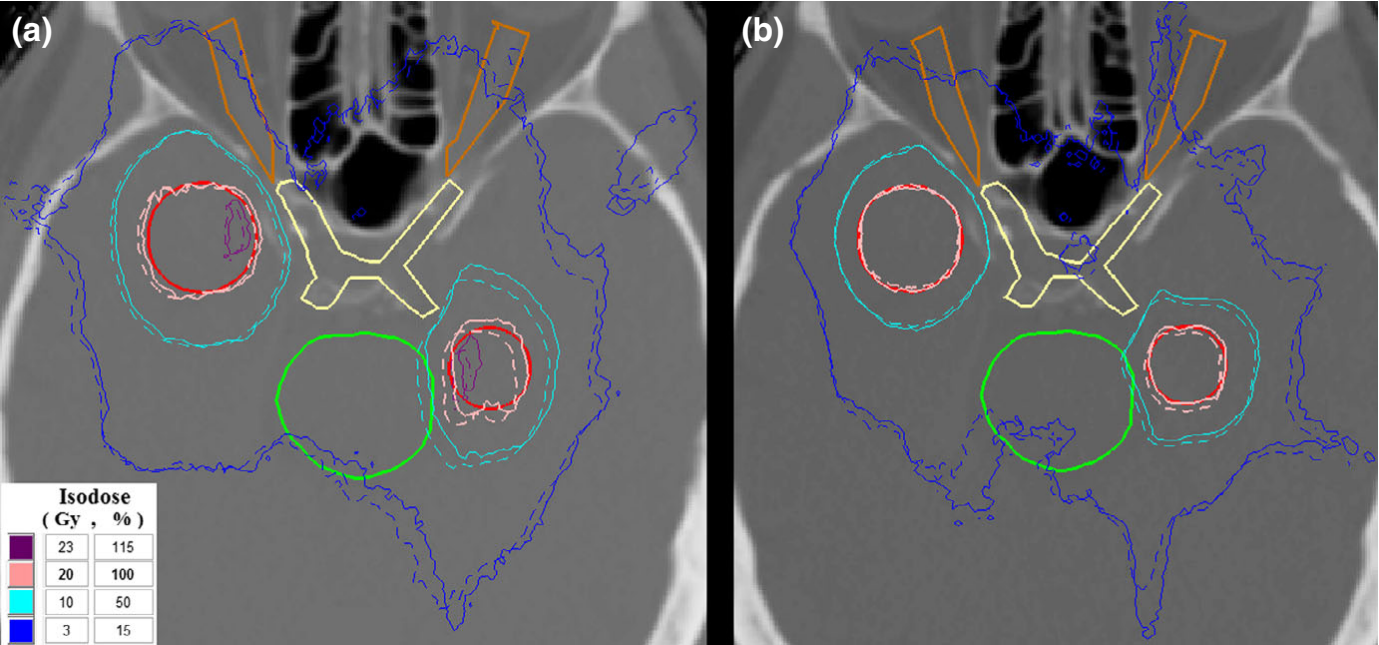
Axial CT slice of patient #5 with isodose lines superimposed for the (a) single‐ and (b) two‐isocenter techniques. Reference dose distributions are represented by solid lines, whereas dashed lines correspond to rotated dose distributions (2°, around all three axes). Contours legend: targets: red, brainstem: green, optic chiasm: yellow, optic nerves: brown.

The dosimetric impact of rotational errors on dose distributions is illustrated in Fig. 3, indicatively for patient #5 and an angular offset of 2° around all three axes. Loss of target conformality to the 20Gy isodose (i.e., the prescription dose) due to the rotation is more evident for the single‐isocenter case [Fig. 3(a)].

**Figure 4:**
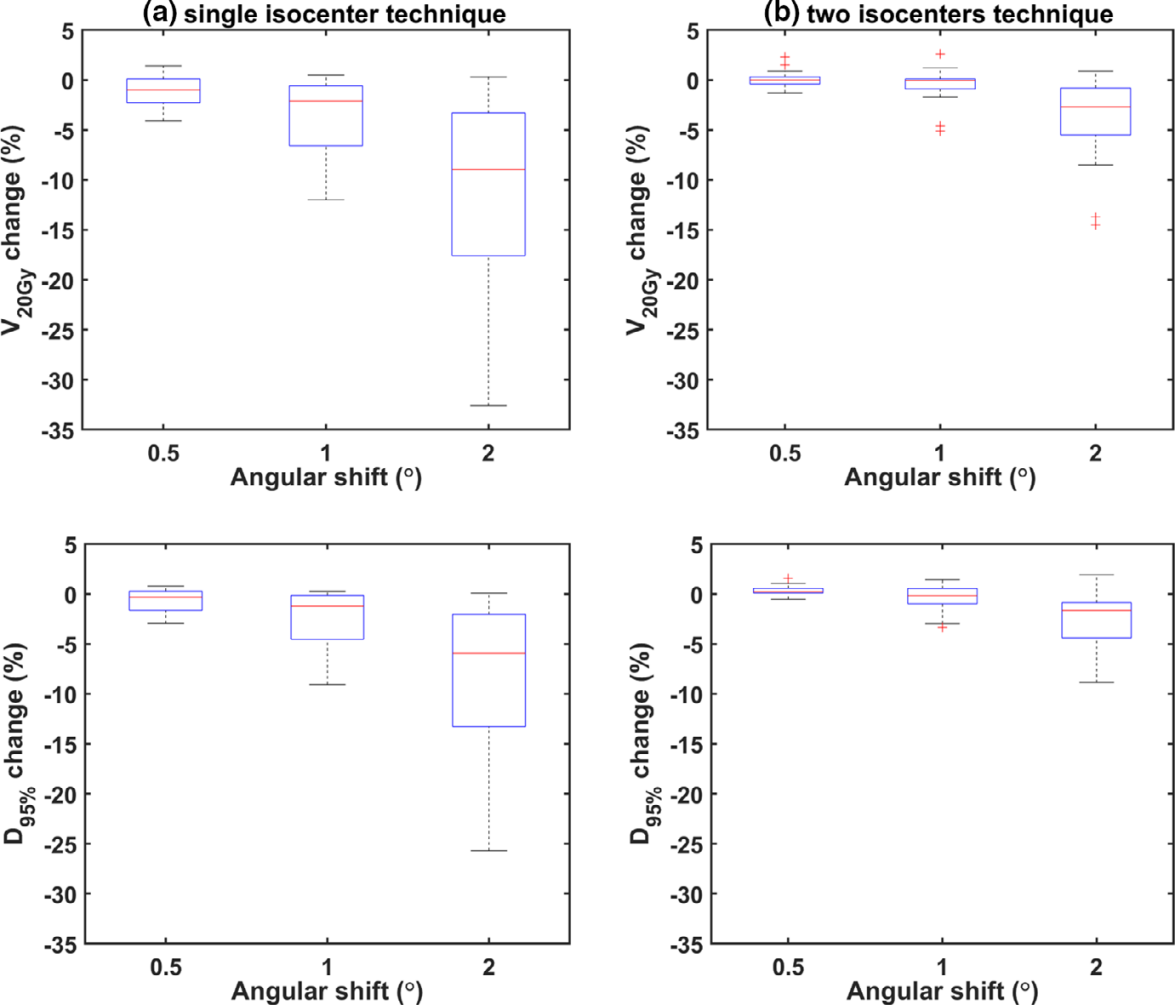
Box and whisker plots summarizing V_20Gy_ (top) and D_95%_ (bottom) deviations induced by simulated rotational errors for the (a) single‐ and (b) two‐isocenter planning techniques. Red lines indicate the median of the data, whereas boxes range from the 1st to 3rd quartile. Whiskers depict the remaining data or extend up to 1.5 times the interquartile range in either direction. Red marks denote any outliers.

The effect is quantitatively represented by the box‐whisker plots shown in Fig. 4 related to changes of target coverage (V_20Gy_) and D_95%_ metrics, induced by rotations of 0.5°, 1° and 2° for all 36 targets. For single‐isocenter plans [Fig. 4(a)], a considerable deterioration (>5%) of V_20Gy_ and D_95%_ in several cases is observed for rotations of 1°. Corresponding deviations for plans utilizing two isocenters [Fig. 4(b)] do not exceed 5%. Even for 2°, the induced effect is considerably reduced, although significant V_20Gy_ and D_95%_ changes (of the order of 10%) were detected.

**Figure 5:**
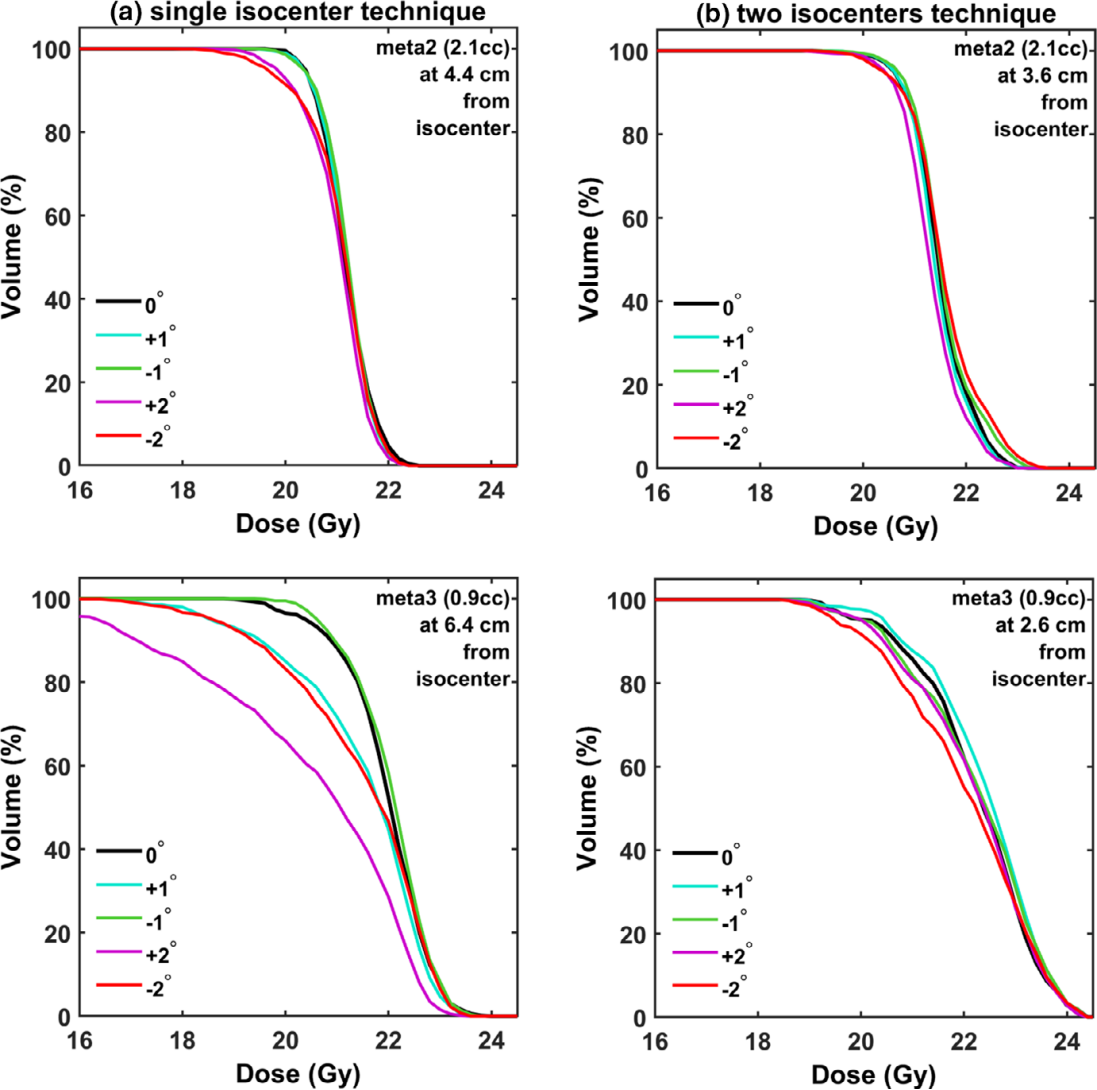
Calculated DVHs for two targets for the (a) single‐ and (b) two‐isocenter planning techniques. Rotations occurred around all three axes.

Results shown in Fig. 4 are characterized by increased spread, in addition to not being normally distributed. Effort was put to correlate the observed underdosage of targets with physical characteristics and, particularly, the lesion volume and distance to nearest isocenter. Indicative results are given in the following figures. In specific, Fig. 5 presents DVHs calculated for a fairly large (2.1cc) and a small lesion (0.9cc, same patient) for both reference plans and certain simulated rotational errors (±1°, ±2°) around the three axes. For the larger target volume, the induced underdosage can be hardly noticed for the two isocenters plan, whereas the effect is increased but still limited for the single‐isocenter plan, even for rotations of 2°. It should be noted that lesion‐to‐isocenter distances were comparable (Fig. 5) for both planning techniques. Contrarily, dosimetric indices for smaller targets were very sensitive to rotational errors. As an instance, in Fig. 5, V_20Gy_ for meta3 dropped to approximately 62% for a rotational offset of 2° and using a single‐isocenter. Even for 1º, corresponding value was 84%. Therefore, resulting target coverage was clinically unacceptable. The effect on the same target is negligible if two isocenters are used and rotational errors do not exceed 1º (Fig. 5).

**Figure 6:**
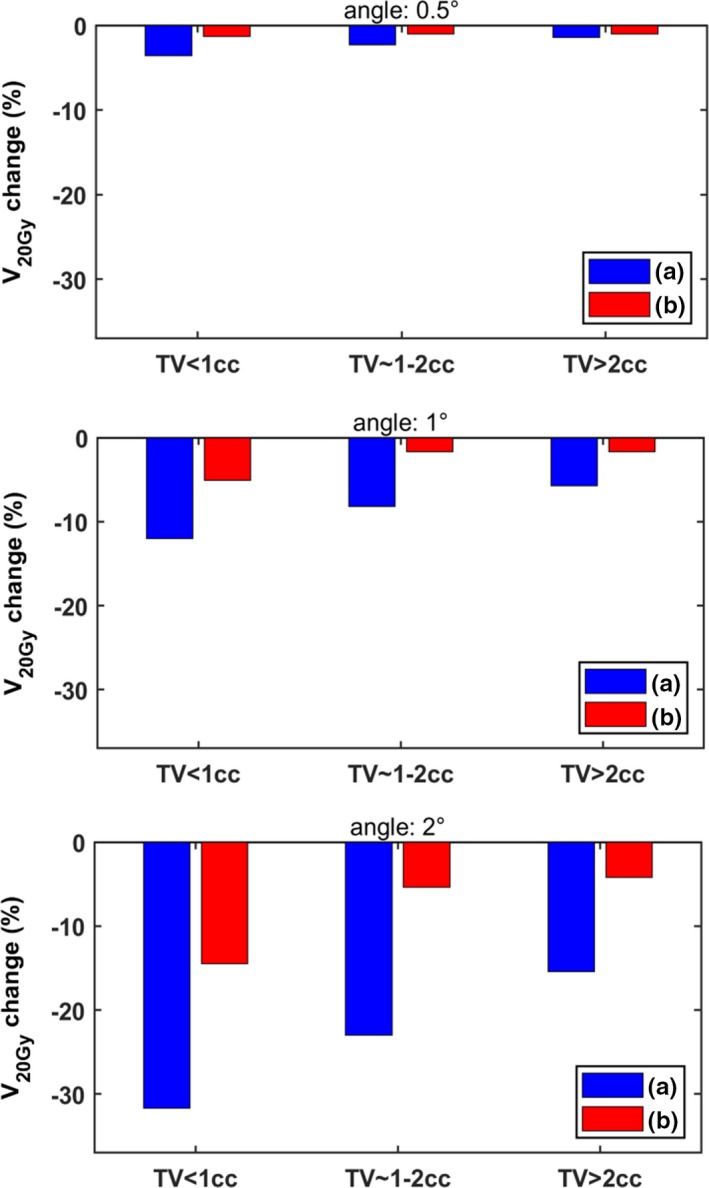
Bar charts of the maximum change in V_20Gy_ are presented for all 36 targets, grouped according to their volume (<1cc, 1‐2cc, >2cc). The bars in blue color are related to the results for (a) single‐isocenter planning technique, whereas the bars in red color are related to the results for (b) two‐isocenter planning technique. Rotations occurred around all three axes.

In an effort to better demonstrate the effect of target size, all 36 lesions were grouped according to their volume (<1cc, 1‐2cc, >2cc) and the maximum change in V_20Gy_ was detected for each group and all rotational errors simulated. Results are presented in Fig. 6. In all cases, the two‐isocenter planning technique is less sensitive to rotational errors. Still, for the smallest targets considered and using two isocenters, V_20Gy_ dropped up to 15% (for an angular offset of 2º), which can be considered clinically unacceptable. Contrarily, for targets larger than 2cc the corresponding maximum detected loss of coverage was limited to 4% (Fig. 6).

**Figure 7:**
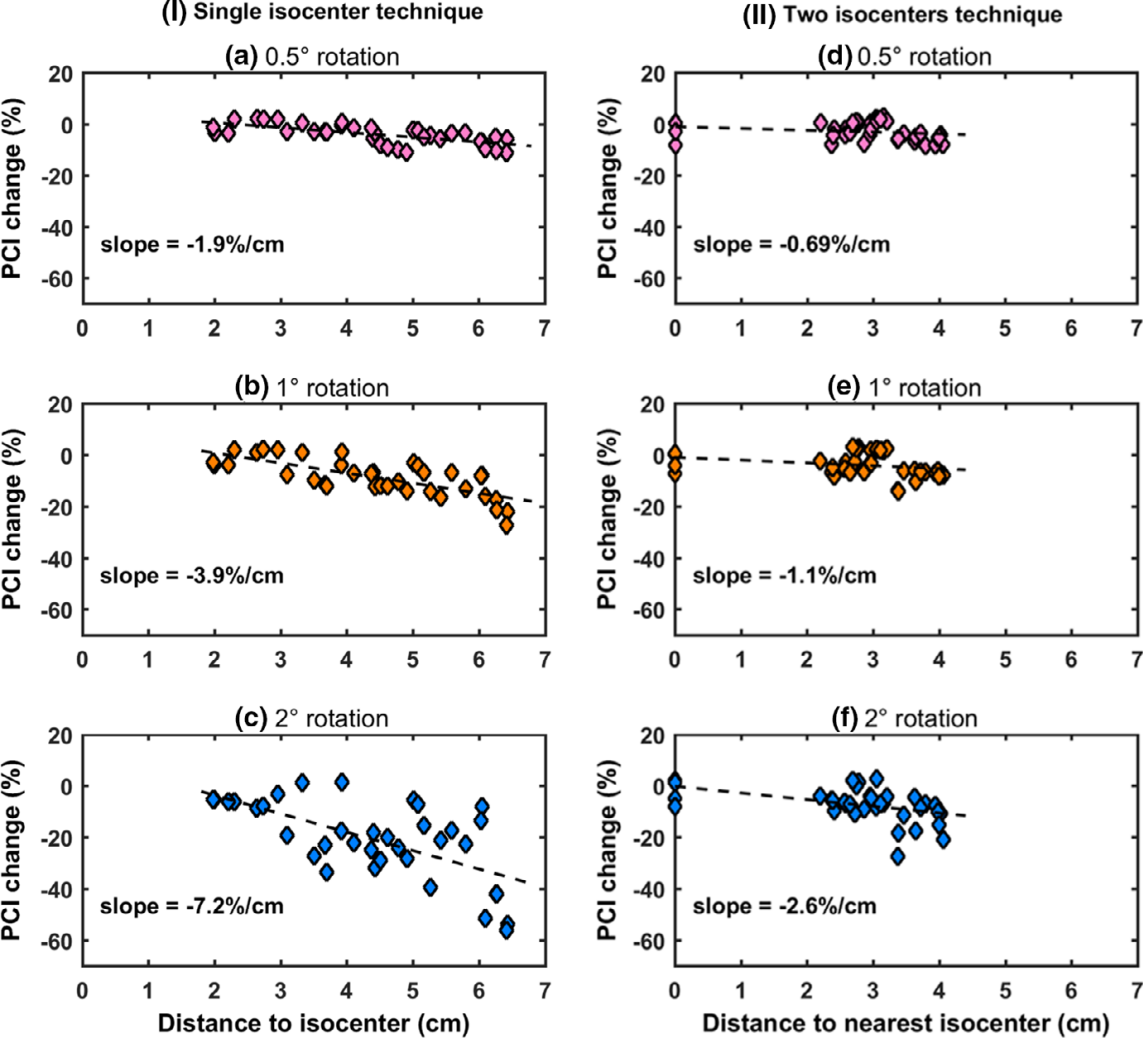
Percentage PCI change plotted against target distance to the nearest isocenter, for all patients and metastases (i.e., a total of 36 targets) considered, for both the (I) single‐ and (II) two‐isocenter techniques and simulated angular offset of 0.5° in (a) and (d), 1° in (b) and (e), 2° in (c) and (f), occurring around all three axes. Fitted dashed trendlines (along with calculated slopes) are also shown. PCI, Paddick’s conformity index.

Dependence of target susceptibility to rotational errors on lesion‐to‐isocenter separation is quantified in Fig. 7. Detected PCI changes (with respect to reference plans) are plotted against distance to the nearest isocenter for both planning techniques. A fitted linear trendline is also given. Indicatively, for the worst case of a 2º rotation, PCI drops by up to 7.2 %/cm [Fig. 7(c)]. Fitted slopes were always steeper by a factor of at least 2.8 (maximum factor of 3.5) for the single‐isocenter plan [Figs. 7(a), (b), (c)] with respect to the two isocenters approach [Figs. 7(d), (e), (f)].

**Figure 8:**
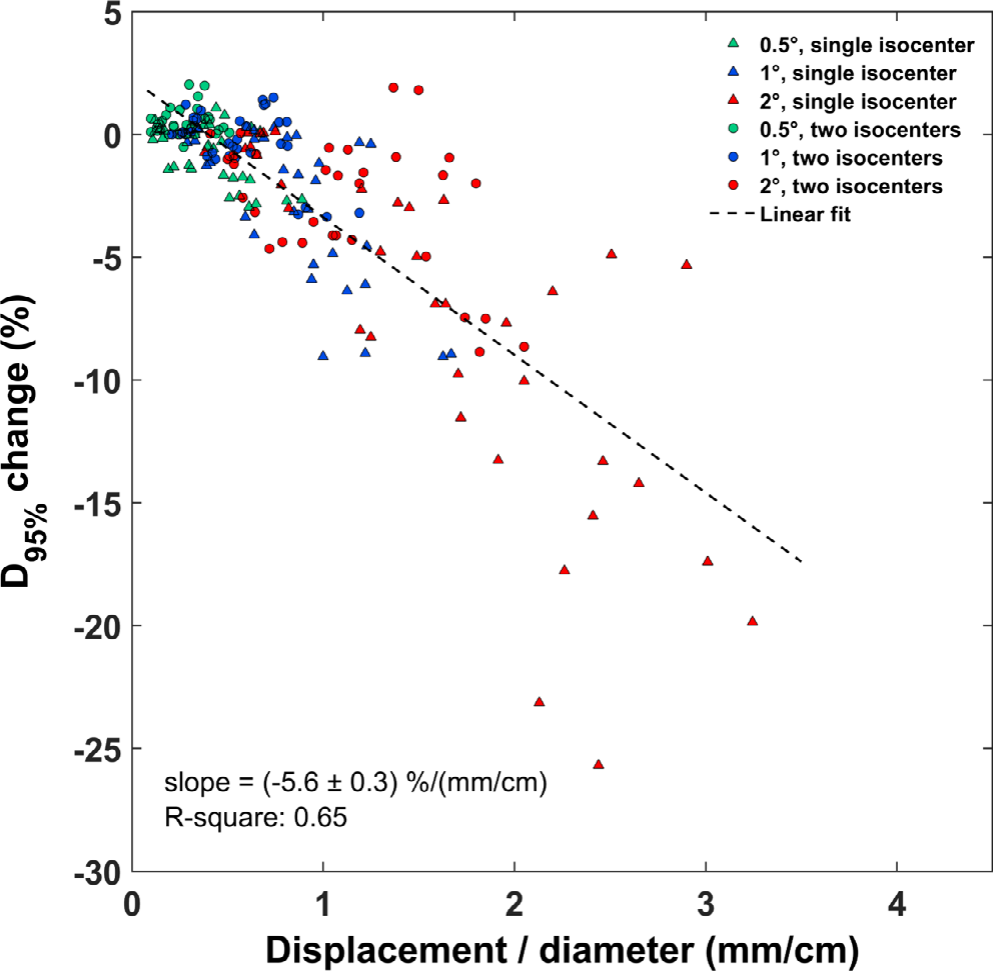
Percentage D_95%_ changes are plotted against the ratio of target‐displacement to target‐diameter, for all patients and metastases (i.e., a total of 36 targets) considered, and stratified by the degree of rotational error (with different marker color) and the isocenter technique (with different marker shape). Rotations occurred around all three axes. A fitted dashed trendline (along with calculated slope, and R‐square) is also shown.

According to the results presented earlier [Figs. 5, 6, 7], lesion size and distance to isocenter are two factors governing the impact of rotational errors on target underdosage. Since the induced target displacement (see Table 4) is the combined geometric effect of an angular offset at the given distance from the isocenter, the ratio of target‐displacement to target‐diameter can be used to account for all parameters in‐play. This quantity takes into account the increased tolerance of larger target sizes to rotational errors, as shown earlier (Figs. 5, 6). Moreover, this ratio is calculated based on target displacement which can be measured in a more straightforward way, compared to measuring/estimating angular offsets and distances to isocenter. Fig. 8 presents changes to D_95%_ against this ratio, for all cases and angular offsets considered. As expected, results do not depend on whether a single or two isocenters were used, as this is taken into account by the distance to isocenter, found in the nominator. A linear fit to the entire dataset (R^2^ = 0.65), revealed a slope of (−5.6 ± 0.3) %/(mm/cm). Furthermore, a calculation of the Pearson coefficient resulted to a statistically significant correlation (*P*‐value < 0.01).

GI was not considerably affected by rotational errors, regardless of the planning technique considered, as percentage changes were in average less than 5%, for any simulated degree or axis of rotation.

#### 3.2.3 Dosimetric effect on OARs

Regarding OARs lying in the vicinity of targets, maximum doses either increased or decreased depending on the magnitude, direction and axis of rotation assumed, as well as relative locations of neighboring targets. As an instance, in [Fig. 3(a)] a rotation of 2° resulted in the 10Gy isoline being shifted in the brainstem, whereas the same isoline (related to another target) moved away from the optic chiasm. However, for the vast majority of cases, axes and angle of rotation, D_max_ and D_0.02cc_ values severely increased, whereas dose reductions occurred rarely. Sign, magnitude, and axes of rotations resulting in additional OAR‐sparing was not definite among the patient cohort and OARs, as the effect is mainly related to the relevant orientation and axis between the structure of interest and the proximal target. In accordance to target‐related results, the magnitude of the effect is also associated with distance to the nearest isocenter.

Table 5 lists percentage changes of dose‐volume metrics considered clinically significant, induced by the simulated rotational errors with respect to values of the reference plans, regardless of the axis and sign of rotation. Since results are not expected to follow the normal (gaussian) distribution, Table 5 presents only the median and range of detected changes. Interestingly, in all OARs and angles, induced maximum and median changes were always positive, that is, corresponded to compromised OAR‐sparing. Moreover, apart from the brain parenchyma, all other OARs were found extremely sensitive to rotational errors. As an example, D_0.02cc_ delivered to the brainstem can increase up to 12.3% (median 7.1%) for angular offsets of 0.5° for the single‐isocenter plan (Table 5). Corresponding values in the case of two isocenters are significantly lower (3.9% and 1.1%, respectively). The most extreme deviation recorded was 63% for a rotation of 2° and one isocenter. In this case, even if two isocenters are employed, exceeding dose to the brainstem was still unacceptable (25.5%). Nevertheless, in all cases and OARs, the induced dosimetric impact was partly mitigated if two isocenters were employed (maximum deviation <10% relative to the reference plan for rotational errors up to 1° around any axis).

**Table 5:** The maximum and median deviations (with respect to reference plans) for clinically used dose‐volume metrics for all patients and OARs considered and all three simulated angles, irrespective of the axis and sign of rotational error assumed. Results are presented for the (a) single‐ and (b) two‐isocenter planning techniques, to assist comparison.

OAR	Metric	Magnitude of rotation (°)	Maximum change (%)		Median change (%)	
			(a)	(b)	(a)	(b)
Brainstem	D_max_	0.5	10.8	5.1	5.3	3.5
		1	20.7	7.0	17.4	5.3
		2	47.2	17.9	32.1	10.7
	D_0.02cc_	0.5	12.3	3.9	7.1	1.1
		1	28.9	10.1	14.5	2.4
		2	63.0	25.5	33.3	13.7
Optic Chiasm	D_max_	0.5	13.2	10.8	6.3	0.0
		1	13.2	10.0	6.3	0.0
		2	55.0	28.6	10.4	6.1
	D_0.02cc_	0.5	5.1	5.2	2.4	0.9
		1	11.9	8.9	5.7	1.9
		2	31.9	14.4	12.9	5.3
Optic Nerve	D_max_	0.5	5.8	2.6	0.0	0.0
		1	25.7	5.9	2.6	0.0
		2	50.0	8.8	10.9	6.2
	D_0.02cc_	0.5	4.7	3.3	1.6	1.7
		1	25.1	9.4	7.0	2.2
		2	51.1	9.9	13.2	8.9
Lens	D_max_	0.5	2.5	0.2	0.0	0.0
		1	16.7	9.8	9.1	2.3
		2	33.3	20.0	13.0	13.0
	D_0.02cc_	0.5	5.2	3.0	4.2	1.8
		1	22.4	9.8	11.1	3.3
		2	28.9	20.7	23.8	17.1
Brain parenchyma	V_7Gy_	0.5	0.9	0.8	0.3	0.2
		1	0.9	0.8	0.7	0.3
		2	1.0	0.9	0.9	0.6
	V_12Gy_	0.5	0.7	0.3	0.1	0.1
		1	1.1	0.4	0.4	0.2
		2	1.1	0.6	0.6	0.6
	V_13Gy_	0.5	0.3	0.3	0.0	0.0
		1	0.7	0.7	0.7	0.0
		2	1.0	0.7	0.7	0.1

According to the results presented in Table 5, compromised OAR‐sparing can occur if rotational errors are not accounted for. The increased dose delivery to critical organs in several cases resulted in dose‐volume indices exceeding the original dose constraints considered during reference treatment planning (see Table 2), that is, rotated plans could be considered clinically unacceptable, even for rotations of 0.5° in a few cases. However, it should be noted that for all angles of rotations investigated, violation of the dose constraints occurred less frequently and to a lesser degree if two isocenters were used.

## 4 DISCUSSION

Overall results of this work suggest that the degree and direction of rotational errors, as well as the distance to nearest isocenter could considerably impact the efficiency of VMAT‐SRS treatments of multiple brain metastases. Treatment techniques employing a single isocenter are more sensitive to rotational errors for both target coverage and OAR‐sparing.

Regarding target dosimetry, other studies have also drawn similar conclusions.^17^, ^29^, ^30^, ^31^, ^38^ More specifically, according to the findings of Guckenberger et al., setup rotational errors of (1.7 ± 0.8)º were detected and corrected for using a six DOF robotic couch.^31^ Still, post‐treatment imaging revealed geometric offsets of (0.9 ± 0.6) mm, suggesting considerable intrafractional patient motion. Such errors were related to reduced target coverage by >5% in 14% of the patients included in the analysis. In another study, for isocenter‐to‐lesion distances up to 75 mm, intrafractional patient positioning uncertainties of up to 1.8 mm were calculated even if a robotic couch is used.^29^ Recently, using kV imaging, it was shown that intrafractional motion is not statistically correlated with treatment duration and can exceed 1.5 mm for cranial multitarget SRS.^28^ In a simulation study involving two metastases per case and one isocenter, rotational errors were introduced around all axes simultaneously.^17^ For errors of 0.5º, D_95%_ and V_95%_ values for all cases were found >95%. Briscoe et al. also investigated cases with two brain metastases using one isocenter.^38^ Loss of target coverage was associated with increasing distance from the isocenter. However, only one target was located at a distance of more than 4 cm, whereas the effect was not studied for more than two lesions per case. Stanhope et al. additionally reported that optimal conformity and gradient indices are achieved when the lesions are located within close proximity to the isocenter and quantified the effect with respect to distance to isocenter, with or without the use a six DOF couch.^30^ The effect was more pronounced for smaller targets (<1cc). In this study, the impact of rotational errors on target dosimetry was studied under a comparative perspective between single‐ and two‐isocenter treatment planning methods. Induced median spatial offsets were reduced by at least 35% if the latter approach is considered. Based on V_20Gy_ and D_95%_ results [Figs. 4 and 6], it is implied that 1° rotational errors are not tolerated in the case of a single isocenter, especially if targets are located several centimeters (typically> ~4 cm) from the isocenter (Fig 7). This remark is in‐line with the recommendations of Briscoe et al..^38^ Using an additional isocenter all targets lay at distances < 4 cm from the nearest isocenter and corresponding plans were found to be less sensitive to rotational errors, with angular offsets of 1° generating clinically acceptable dose distributions. However, reduced lesion‐to‐isocenter separations may result in minimizing target displacement but smaller target sizes are still very sensitive to rotational errors (Fig. 6). The dosimetric impact can be clearly correlated with the target‐displacement to target‐diameter ratio, as shown in Fig. 8, which is independent to the planning technique employed.

To the best of our knowledge, no studies have evaluated the dosimetric effect of rotational errors on OARs for multitarget intracranial SRS. Peng et al. simulated angular offsets only for single‐target fractionated SRS and reported exceedance of tolerance doses in OARs lying in the vicinity of the target, with the most dramatic increase detected for the brainstem, even for a rotation of 1°.^24^ Based on the results of this study (Table 5), rotation tolerances in multitarget single‐isocenter applications are more stringent, as — in a few cases — plans that could be considered clinically unacceptable were obtained even for rotations of as low as 0.5º. If two isocenters are employed, increase in dose to OARs is substantially reduced (Table 5) but plans that could be considered unacceptable were still noted, although rarely. Therefore, if OARs are located in the vicinity of targets, these remarks should be taken into consideration when employing single‐ or two‐isocenter VMAT‐SRS for the treatment of several lesions, especially if a six DOF couch is not available.

In an effort to reassure target coverage in VMAT‐SRS for multiple brain metastases, several studies have proposed either tolerance levels for rotational uncertainties or, alternatively introduction of safety margins during treatment planning.^29^, ^30^, ^31^, ^38^ More specifically, Briscoe et al. established a threshold of as low as 0.5°.^38^ Stanhope et al. suggested a margin of 0.35 mm per centimeter of distance to isocenter, in the case a six DOF couch is not available.^30^ If rotational setup errors are detected and corrected for, this margin can be reduced to 0.1 mm/cm.^30^ Another study suggested that target coverage is assured if a margin of 2 mm is applied.^47^ Regarding intrafractional motion, a 1‐mm margin was proposed.^31^ However, none of these recommendations take into account OAR‐sparing. As an instance, margins of just a few millimeters result in an increase in dose to normal brain parenchyma and, consequently, increased risk of radiation‐induced brain necrosis.^48^, ^49^, ^50^ Furthermore, according to the results of this study, tolerance levels of rotational errors related to OAR‐sparing (e.g., maximum dose to brainstem, Table 5) are more stringent compared to considering target coverage alone. As an alternative or complement to the introduction of margins, limiting lesion‐to‐isocenter distances (using additional isocenter(s)) appears to also mitigate the induced dosimetric effect.

A number of limitations of this study are noteworthy. The single‐ and two‐isocenter reference plans were similar and clinically acceptable but not identical in terms of plan quality. Using a second isocenter resulted in slightly — yet systematically — superior plans, as expected.^20^ Although this approach allows for comparison between planning techniques (i.e., the main focus of this study), the dosimetric advantage of using two isocenters to mitigate rotational errors would have been clearer in case identical plans were used as references. Furthermore, rotated dose distributions were obtained by applying an angular shift to the corresponding reference ones. Performing dose re‐calculations within rotated patient geometries would result in more accurate dose distributions, although such approach would also require heavy computational time and effort. However, it is geometrically expected that small angular shifts will not induce a considerable change to the depth of a target with respect to the beam paths, especially for cranial cases where the patient’s external surface varies smoothly. Therefore, the dosimetric effect of this approximation can be considered negligible which was also confirmed in the work of Roper et al.^17^ Another limitation of this study is that presented results depend on the given spatial distribution, size and shape of targets included in the analysis. Investigation of larger (>4cc) target volumes was not performed, although it has been shown that the induced dosimetric effect could also vary accordingly.^24^ The maximum lesion‐to‐isocenter distance included in this analysis was limited to 6.5 cm. However, in single‐isocenter SRS treatment planning, larger separations can be encountered, exceeding 10 cm in a few cases.^17^, ^30^ In addition, up to four targets per patient were studied, despite the fact that the number of lesions considered treatable by SRS has recently increased.^4^, ^5^ Assuming a larger number of metastases per case would result in increased average lesion‐to‐isocenter distances, and tolerance of rotational uncertainties is expected to be much more stringent in such distant targets and adjacent OARs. Therefore, presented results should be regarded as indicative for cases similar to the ones presented herein.

Image guidance and a stringent patient setup protocol are essential in order to minimize potential setup errors in single‐isocenter multitarget VMAT‐SRS. In absence of a six DOF couch and for patients with three or more brain metastases and adjacent OARs, limiting lesion‐to‐nearest isocenter distance to ~ 4 cm (by introducing additional isocenter(s)) appears to be a safe‐side treatment planning approach that partly mitigates the effect on targets and OARs. However, in specific cases and orientations of rotational errors, plans that could be considered clinically unacceptable were obtained even for angles of as low as 0.5°. Despite that the effect was rare for plans of two isocenters, still, OAR‐sparing cannot be guaranteed. Employing a six DOF robotic couch could minimize the required margins and/or number of isocenters, although intrafractional patient motion still remains a concern.^26^, ^30^, ^31^ Future work will focus on the investigation of the overall spatial uncertainties and corresponding additional margins required, incorporating MR‐related distortions, MR/CT image registration accuracy and mechanical dose delivery uncertainties.^32^, ^33^, ^34^, ^35^, ^51^

## 5 CONCLUSION

This simulation study focused on rotational errors (mainly stemming from either the setup procedure or intrafractional patient motion) in multitarget intracranial VMAT‐SRS, under a comparative perspective between single‐ and two‐isocenter planning approaches. Considering target dosimetry alone, rotations of 1° generate clinically acceptable dose distributions if two isocenters are used, whereas for the single‐isocenter technique angular offsets should not exceed 0.5°. Limiting the lesion‐to‐isocenter distances to ~4 cm (by introducing additional isocenter(s)) appears to partly mitigate severe target underdosage. However, smaller target sizes (especially < 1cc) may still exhibit increased sensitivity to rotational errors. In any case, lesion size and distance to isocenter are two factors governing the impact of rotational errors on target underdosage and thus the later was found to be clearly correlated with the target‐displacement to target‐diameter ratio, a factor that takes into account the increased sensitivity of smaller target sizes to rotational errors. Moreover, if OAR‐sparing is also a concern (i.e., OARs lying in close proximity to targets), more stringent tolerances apply. Plans that could be considered clinically unacceptable were obtained for both planning techniques even for the smallest angular offset considered (0.5°), although the effect was rarer for plans involving two isocenters, as the resulting dose‐increase to OARs was limited compared to the single‐isocenter approach.

## CONFLICTS OF INTEREST

None.
